# Associations between area socioeconomic status, individual mental health, physical activity, diet and change in cardiometabolic risk amongst a cohort of Australian adults: A longitudinal path analysis

**DOI:** 10.1371/journal.pone.0233793

**Published:** 2020-05-29

**Authors:** Suzanne J. Carroll, Michael J. Dale, Theophile Niyonsenga, Anne W. Taylor, Mark Daniel

**Affiliations:** 1 Australian Geospatial Health Laboratory, Health Research Institute, University of Canberra, Australian Capital Territory, Canberra, Australia; 2 Health Research Institute, University of Canberra, Australian Capital Territory, Canberra, Australia; 3 Discipline of Medicine, The University of Adelaide, Adelaide, South Australia, Australia; 4 Department of Medicine, St Vincent’s Hospital, The University of Melbourne, Fitzroy, Victoria, Australia; University of Turku, FINLAND

## Abstract

Presumed pathways from environments to cardiometabolic risk largely implicate health behaviour although mental health may play a role. Few studies assess relationships between these factors. This study estimated associations between area socioeconomic status (SES), mental health, diet, physical activity, and 10-year change in glycosylated haemoglobin (HbA_1c_), comparing two proposed path structures: 1) mental health and behaviour functioning as parallel mediators between area SES and HbA_1c_; and 2) a sequential structure where mental health influences behaviour and consequently HbA_1c_. Three waves (10 years) of population-based biomedical cohort data were spatially linked to census data based on participant residential address. Area SES was expressed at baseline using an established index (SEIFA-IEO). Individual behavioural and mental health information (Wave 2) included diet (fruit and vegetable servings per day), physical activity (meets/does not meet recommendations), and the mental health component score of the 36-item Short Form Health Survey. HbA_1c_ was measured at each wave. Latent variable growth models with a structural equation modelling approach estimated associations within both parallel and sequential path structures. Models were adjusted for age, sex, employment status, marital status, education, and smoking. The sequential path model best fit the data. HbA_1c_ worsened over time. Greater area SES was statistically significantly associated with greater fruit intake, meeting physical activity recommendations, and had a protective effect against increasing HbA_1c_ directly *and* indirectly through physical activity behaviour. Positive mental health was statistically significantly associated with greater fruit and vegetable intakes and was indirectly protective against increasing HbA_1c_ through physical activity. Greater SES was protective against increasing HbA_1c_. This relationship was partially mediated by physical activity but not diet. A protective effect of mental health was exerted through physical activity. Public health interventions should ensure individuals residing in low SES areas, and those with poorer mental health are supported in meeting physical activity recommendations.

## Introduction

The prevalence of cardiometabolic disease (CMD) such as cardiovascular disease (CVD; i.e., all diseases of the heart and blood vessels including coronary heart disease and stroke) and type 2 diabetes mellitus (T2DM) continues to rise worldwide, presenting a substantial challenge to public health [1, 2]. Though there is some suggestion that trajectories of age-standardised CVD prevalence rates have plateaued, even declined in some countries, these rates remain high and may further rise given increases in obesity and T2DM, both key risk factors for CVD [1, 3–5]. The Organisation for Economic Co-operation and Development (OECD) estimate CMD represents a substantial portion (approximately 40%) of the health burden in OECD countries [6].

Various individual-level factors are well-established as linked to CMD and cardiometabolic risk (CMR). These include sociodemographic factors such as older age, male sex, and lower socioeconomic status (SES), particularly education [7, 8], along with behavioural factors including smoking, poor diet and insufficient physical activity [9–13]. Elevated levels of physical activity are protective against all-cause mortality [13], and CVD events and mortality [12]. Greater fruit and vegetable intake (up to a limit of 5 serves per day) reduces risk of CVD mortality [11]. Diets low in fruits and vegetables and high in unhealthful food, along with low levels of physical activity are, however, highly prevalent [14–16]. Given the established benefits of compliance with physical activity and diet recommendations, these behaviours are often targeted by interventions intending to improve population health.

Mental health has also been implicated in the development of CMD [8]. Mood disorders have been linked to the development of a variety of physical conditions including heart disease, stroke, hypertension, and T2DM [17]. [18–22] Symptoms of depression include changes in appetite (increased or decreased), energy and motivation (both decreased) which can influence diet and physical activity participation [22, 23]. Poor mental health is associated with disordered eating, unhealthful dietary preferences, and physical inactivity [20]. Thus, poorer mental health may directly influence CMR or act through behavioural factors such as poor diet and physical inactivity though this has rarely been assessed.

Putting these individual-level relationships into context, features of residential environments are known to predict mental health, diet, physical activity, CMD and CMR. Residing in a low SES area is well established as related to adverse health outcomes including obesity, hypertension, diabetes, dyslipidaemia, and the metabolic syndrome [24]. Similarly, studies have reported that living in a low SES area is related to lower physical activity level and poor diet [25–28]. Not all environment-behaviour studies align, however, as some studies, particularly those investigating low area-SES and diet [e.g., 29, 30] have reported null relationships. Regardless, it is generally assumed that health behaviours provide a link between area SES and health outcomes, including CMD.

Mental health may also play a role in linking area SES and health outcomes [31]. A recent non-systematic narrative review reported a host of associations between low area SES and poorer mental health (psychological distress, depression and suicide) [32]. Indeed residential areas likely influence mental (and physical) health through both structural (e.g., area SES, residential stability, built environment) and social (e.g., neighbourhood disorder, social cohesion, social ties with neighbours, crime, violence, graffiti) processes [33, 34]. Areas characterised by a poor quality environment (e.g., lack of health-related resources, greater exposure to noise, disorder, violence and trauma, poor social ties and lack of social cohesion) can increase social strain and psychosocial stress, consequently increasing risk for poor mental health and depression [33, 35, 36]. Mental health may then be directly linked to CMR through physiological responses such as allostatic loading or through behavioural responses such as poor health behaviours which may then feed back in a recursive loop to poorer mental health [36]. However, poor area SES may also be linked directly to adverse physiological responses and health outcomes through a non-cognitive pathway (i.e., not consciously perceived by the individual) and thus not be mediated through mental health or health behaviour [for further discussion see 36].

The links between areas, mental health, health behaviour and health outcomes are likely highly complex, functioning through webs of interacting pathways [36, 37]. The network of pathways from more distal environmental influences through to proximal psychosocial and behavioural risks and physical health outcome is therefore likely to consist of many factors acting either simultaneously or sequentially [36]. For example, area SES may directly influence CMR, may impact mental health, diet and physical activity, each of which then influences CMR (i.e., parallel mediation), or, area SES may influence mental health which in turn influences health behaviours and thus CMR (sequential mediation). However, there is a lack of research assessing these complex pathways.

Few studies have empirically tested mediating pathways from environmental exposures to health outcomes, though reviews have repeatedly called for such investigations [24, 38]. Explicit testing of mediating pathways is necessary to support causal inference by providing evidence of biological plausibility for the link between environments and health [36, 39]. Those studies that have empirically tested mediating pathways have typically focused on few potential intermediary factors simultaneously and have used a parallel mediation structure. Such studies do not account for the complexity of pathways likely linking environments to health. Improving our understanding of this complex web, including the relative contributions of the various pathways, is necessary to improve intervention design and thus intervention effectiveness. Moreover, a longitudinal approach to testing mediating pathways is necessary to provide stronger evidence of actual mechanisms of how environments shape physical health. Masses of cross-sectional studies that account neither for time nor mediation effects cannot, by definition, support causal inference yet are consistently applied not only to guide interventions, but as a basis for public health policy [e.g., 40]. Longitudinal studies are increasingly called for to provide evidence of causal effects [24, 33, 36, 38]. This longitudinal study used path analysis to estimate the longitudinal associations between area SES, mental health, diet and physical activity, and change in HbA_1c_, including assessment of parallel and sequential mediation path structures, in a cohort of Australian adult city-dwellers.

## Materials and methods

This longitudinal observational study was part of the Place and Metabolic Syndrome (PAMS) Project which investigated links between residential environmental features and cardiometabolic risk. PAMS received ethics approval from the University of South Australia (P029-10 and P030-10), Central Northern Adelaide Health Service (Queen Elizabeth Hospital; Application No. 2010010), and the South Australian Department for Health and Ageing (Protocol No. 354/03/2013 and HREC/13/SAH/53) Human Research Ethics Committees. PAMS used cohort data from the North West Adelaide Health Study (NWAHS). Written informed consent of NWAHS cohort participants was obtained prior to each wave of data collection.

The NWAHS was a population-based biomedical cohort of randomly selected adults (18 years and older) residing in the northern and western regions of metropolitan Adelaide, the capital of South Australia [41]. In 2001 (cohort baseline), these regions accounted for 38% of Adelaide’s 1.1 million population [42]. The NWAHS included three waves of clinical data collected over 10 years: Wave 1 (2000–03, n = 4056), Wave 2 (2005–06, n = 3205, 79% of baseline sample) and Wave 3 (2008–10, n = 2487, 61.3% of baseline sample). For the current study, a geographic information system (GIS) was used to spatially join NWAHS data with 2001 Australian Census data [43].

## Cohort participants

Households within the NWAHS region (defined by postcode) were randomly selected from the Australian Electronic White Pages telephone directory. The resident adult (18 years or over) who most recently had their birthday was asked to participate in the study. Computer-Assisted Telephone Interviews (CATIs) and self-reported questionnaires were used at each NWAHS wave to collect residential address, sociodemographic, behavioural and health-related information. The participant residential address was used to create a geo-reference enabling spatial linkage with census data. Additional information on the NWAHS is available elsewhere [41, 44]. Cohort participants with addresses that could not be geocoded (n = 15), who resided outside of the PAMS urban area (n = 154), moved between waves (n = 909), resided in a suburb with fewer than 5 participants (n = 21), lacked baseline covariate data (n = 110), identified as having CVD or T2DM at baseline (i.e., HbA_1c_ values ≥ 6.5% (48 mmol/mol), fasting plasma glucose level ≥ 7 mmol/L, or self-reported previous diagnosis by a doctor, n = 507), or lacked at least one wave of HbA_1c_ data, were excluded from analyses. The analytic sample included 2337 participants.

## Measures

### Outcome measure: HbA_1c_

CMR was defined by HbA_1c_ at three timepoints. HbA_1c_ concentration, reflecting 2–3 month time-averaged blood glucose level [45], was assayed from fasting blood samples collected during clinic visits at each data collection wave [41]. HbA_1c_ concentrations of 6.5% or greater are considered indicative of diabetes [46], while cardiovascular disease (CVD) risk rises with increasing HbA_1c_ [47]. Consequently, HbA_1c_ provides a defensible estimate of cardiometabolic risk, (i.e. risk of T2DM or CVD, or both).

### Environmental exposure: Area SES

Area SES was expressed using the Australian Bureau of Statistics (ABS) 2001 Census-based Socio-Economic Index for Areas, Index for Education and Occupation (SEIFA-IEO) defined for State Suburbs [48]. State Suburbs are formed by aggregating census collection districts to align with the most recently gazetted suburb at the time of the census [49]. Composite SEIFA SES indices, such as the SEIFA-IEO, are commonly used in Australia to represent area-level SES.

### Individual-level psychosocial and behavioural factors

Measures of interest included: 1) mental/emotional health, assessed using the mental health component score (MHCS) of the 36-item Short Form Health Survey(SF-36); 2) dietary intakes (counts of daily fruit and vegetable serves); and 3) physical activity (categorisation of total physical activity time/week). These measures were each collected at Wave 2.

The SF-36 MHCS was used to express participant mental/emotional health. The SF-36 measures participant perceived health-related quality-of-life and includes two summary scores capturing the physical (physical component score; PCS) and mental (MHCS) dimensions of health status [50]. SF-36 MHCS was calculated using the structural equation modelling approach validated and recommended by Tucker et al. [51]. The MHCS captures overall function consequent to mental/emotional health [52] and identifies anxiety and depression in both general [53] and clinical populations [54, 55]. The SF-36 MHCS has been previously established as demonstrating adequate reliability (Cronbach’s α = 0.84, [50] and = 0.95 [56]) and validity (correlation to the Emotional Reactions component of the Nottingham health profile of 0.67 [56]).

Self-reported dietary intakes of fruits and vegetables (counts of servings per day) were collected via CATI at Wave 2 using questions derived from the Australian National Health Survey [57]. Participants were provided examples of serving sizes and asked to report the usual number of serves of fruits and vegetables they consumed per day. Diet measures were assessed for distributional problems and different expressions were explored such as categorisation and transformations. The distributions of measures were not so divergent from normal as to be considered problematic in modelling using a robust estimation approach and Monte Carlo integrations for estimating confidence intervals [58].

Self-reported physical activity information was collected using Australian National Health Survey (NHS) questions as part of the Wave 2 paper-based questionnaire. The NHS physical activity questions have moderate test-retest reliability with an intra-class correlation of 0.57 (0.49–0.68) for total minutes of activity and a percentage agreement of classification of activity status (active, insufficiently active, or sedentary) of 59.8% with a Kappa of 0.40 (0.26–0.53) [59]. Captured information included time spent participating in walking, moderate and vigorous physical activity for sport, recreation, or fitness. Physical activity data were prepared per recommendations from the Active Australia Survey [60]. Total time (in minutes) spent doing physical activity was calculated as follows, with the additional health effects of vigorous activity compared to lower intensity levels accounted for within the calculation [60]:

Totaltime=minuteswalking+minutesofmoderatephysicalactivity+2xminutesofvigorousactivity

Where participants had missing physical activity component data, total physical activity was coded missing. Physical activity behaviour was categorised as: 1) meeting recommendations (≥150 minutes/week) versus 2) not meeting recommendations (reference category) [61].

### Covariates

Age, sex, employment status (full-time, part-time, or not in the work force), level of education (university graduate or not), marital status (married/de facto or single), and smoking status (current or non-smoker) were included as covariates. These measures were selected based on previous research regarding area SES, mental health, diet and physical activity behaviour, and health outcomes such as HbA_1c_, and analyses assessing cohort loss to follow-up and data missingness. Their inclusion satisfies the analytic criterion of *missing at random* [62].

### Analyses

**Fig 1:**
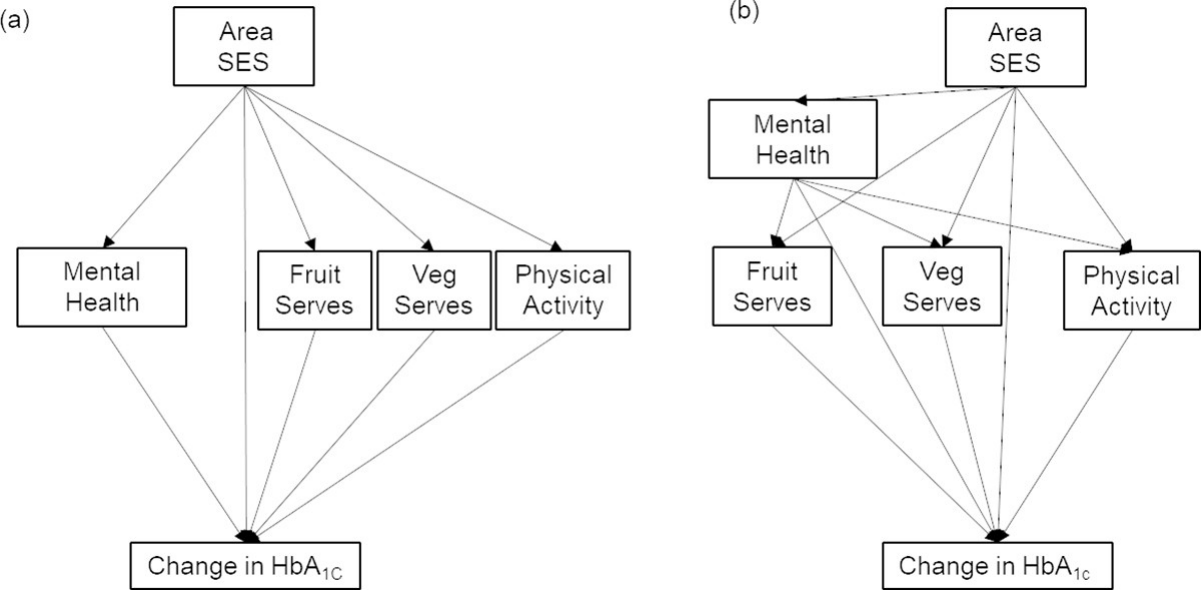
Simplified path model diagrams: a) parallel; b) sequential.

Latent growth models in Mplus (version 8, Muthen & Muthen 1998–2017) with a structural equation modelling (SEM) approach were used to estimate direct and indirect effects based on path diagrams illustrated in Fig 1. This approach allowed the simultaneous estimation of all effects within one model as opposed to using multiple separate regression models [58, 63, 64].

HbA_1c_ trajectories were modelled as latent variables (slope for change over time, and intercept for baseline) with random effects to allow for participant-specific variations. As there were only three waves of data, only linear growth curves were considered [65].

Path models were estimated using full information maximum likelihood (FIML) with robust standard errors and Monte Carlo integration. The FIML approach allows for the inclusion of cases with missing information on mediators [66, 67]. Robust standard errors (Huber-White sandwich estimator) were calculated to account for dependent variable (including mediators) deviations from distributional assumptions [58]. Monte Carlo numerical integration with maximum likelihood estimation is required when the posterior distribution for the latent variables (intercept and slope) does not have a closed-form expression [68, 69]. Model constraints were used to estimate indirect effects as the product of coefficients for continuous measures (both outcome and mediator) and using causal effects formulae for pathways involving physical activity (i.e., a categorical mediator with continuous outcome) [58]. The summative effects of parallel pathways (e.g., the total diet effect being the summed effect of the fruit intake and vegetable intake pathways) were also calculated within MPlus using model constraints.

Two separate path models were estimated: 1) parallel mediation (Fig 1A); and 2) sequential mediation (Fig 1B). These models were compared based on model fit statistics (Akaike’s Information Criteria [AIC] and Bayesian Information Criteria [BIC]) to determine which path model best fit the data. Models accounted for spatial clustering within suburbs. Conventional model fit indices for SEM (e.g., χ^2^, CFI and TLI, RMSEA, SRMR) are not reported as they are not available for complex growth curve models fitted to time-unbalanced longitudinal data accounting for spatial clustering and using Monte Carlo integration (i.e., SEM using RANDOM and COMPLEX within MPlus Version 8.3, Muthen & Muthen, Los Angeles, CA, USA).

Area SES, mental/emotional health, and fruit and vegetable intakes were standardised (i.e., z-scores with a mean of 0 and a standard deviation of 1) prior to inclusion in analytic models to allow for ease of comparison of effects. Physical activity was not standardised due to its categorical nature. Results are reported unadjusted and adjusted for individual-level covariates predicting outcome (latent variables), intercept (baseline), slope (rate of change), and mediators. Statistical significance level was alpha of 5%.

## Results

Characteristics of the sample are presented in Table 1. The mean sample age was 50 years, slightly more than half (55%) of the sample were women, 19% were smokers, and 12% were university educated. Only 56% were employed reflecting the older age of the sample and proportion retired from work. The majority of the sample did not meet health recommendations for physical activity, and fruit and vegetable serving intakes were low. The mean area of suburbs (spatial units for area SES and clustering) was 2.36 km^2^.

**Table 1:** Sample characteristics and area socioeconomic status (SES).

Measure	Mean (SD) or n (%)
Length of follow-up (for those with 3 waves of data) (years)	7.83 (1.03)
Length of follow-up (including individuals with measures only at 1 time-point)	5.47 (3.26)
HbA_1c_ (%, Wave 1) n = 2334	5.41 (0.46)
HbA_1c_ (%, Wave 2) n = 1842	5.55 (0.47)
HbA_1c_ (%, Wave 3) n = 1402	5.68 (5.01)
*Wave 1 measures*:	
Age (years)	49.6 (15.7)
Women	1290 (55.2)
Men	1047 (44.8)
Current smoker	446 (19.1)
Non-smoker	1891 (80.9)
Education (university graduate)	282 (12.1)
Education (less than university educated)	2055 (87.9)
Marital status (married/de facto)	1492 (63.8)
Not married /de facto	845 (36.2)
Employed	1303 (55.8)
Not employed (unemployed, home duties, retired etc)	1034 (44.2)
Area-level SES (SEIFA-IEO) ^1^	943.8 (77.2)
Area of spatial unit (i.e., suburb, n = 121) km^2^	2.36 (2.54); median 1.61 (IQR 1.05–2.72)
*Wave 2 (mediation) measures*:	
SF36 MHCS	50.2 (10.0)
Fruit intake (daily count of servings)	1.5 (1.0); median 1 (IQR 1–2)
Vegetable intake (daily count of servings)	2.5 (1.4); median 2 (IQR 1–3)
Does not meet PA recommendations	900 (59.9)
PA meets recommendations	603 (40.1)

^1^ raw data reported, i.e., not standardised to a mean of 0 and SD of 1; Abbreviations: HbA_1c_, glycosylated haemoglobin; PA, Physical Activity; SES, Socio-Economic Status; SEIFA-IEO, Socio-Economic Index for Areas, Index for Education and Occupation; SD, standard deviation; SF36 MHCS, Short Form 36 Mental Health Component Score.

Latent variables representing the intercept and slope for change in each outcome over time were estimated using growth models with no predictors. Estimated latent variables were statistically significant with the estimated slopes indicating that HbA_1c_ increased (worsened) over time (β 0.035% points per year [95%CI: 0.029, 0.041], p<0.001). Accounting for individual-level covariates increased the estimated latent variable slope (to 0.048% points per year [0.036, 0.061], p<0.001).

**Fig 2:**
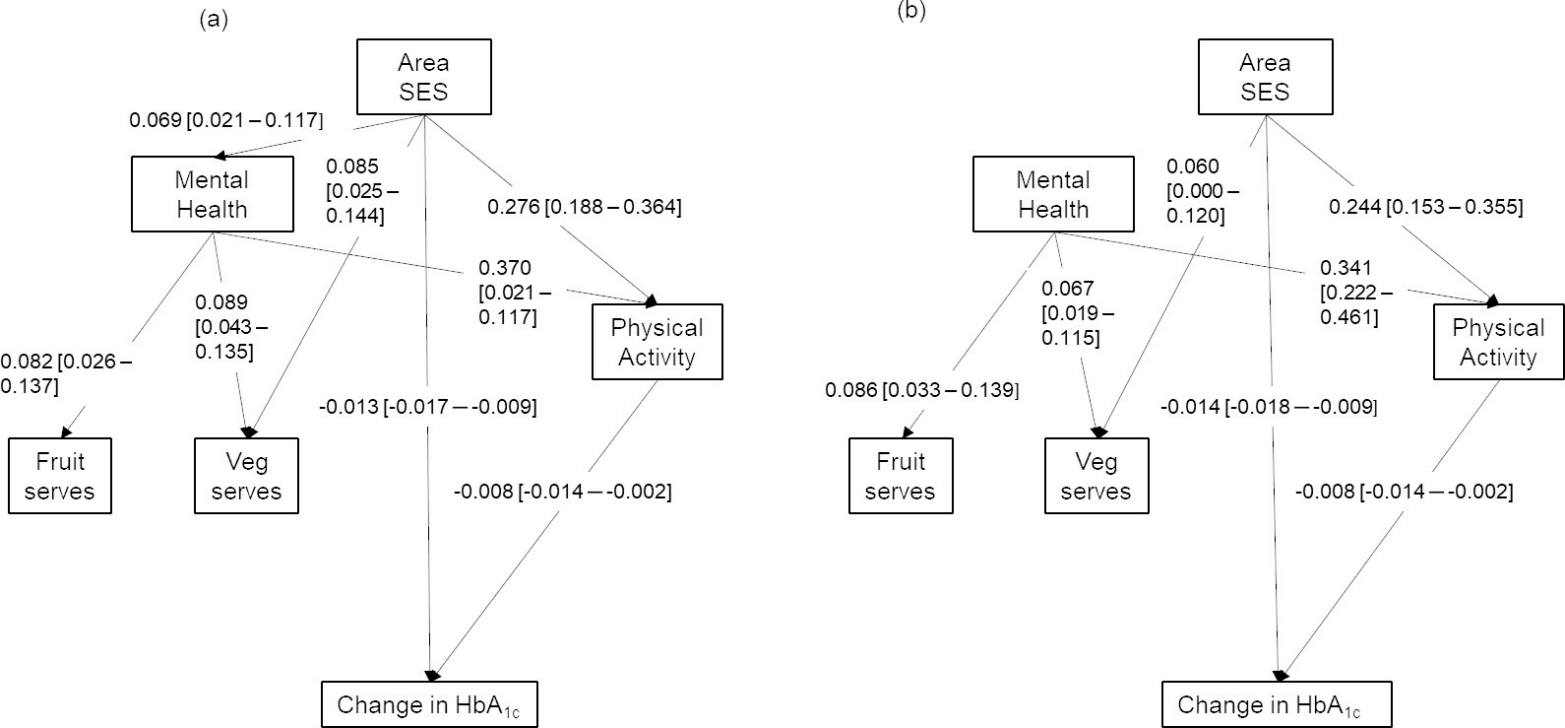
Results of unadjusted (a, left) and adjusted (b, right) sequential mediation path models (only statistically significant pathways shown on diagram).

Both parallel and sequential mediation path models were performed as defined in Fig 1A and 1B. Model fit statistics (models including covariates) indicated sequential mediation models including covariates had the best fit (parallel mediation model: AIC = 21774.984, BIC = 22126.138; sequential mediation model: AIC = 21729.326, BIC = 22096.750). In addition, sequential mediation from area SES to health behaviour then mental health (i.e., the reverse mediation paths of those shown in Fig 1B) and change in HbA_1c_ was assessed (including covariates: AIC = 21737.499, BIC = 22105.923) but model fit for the original hypothesised paths (Fig 1B) was superior. Only sequential model estimates (Fig 1B, Table 2) are reported here. Parallel mediation results are provided in S1 File of Table 1 (see S1 File). To ease interpretation and for clarity of reporting, only those pathways that were statistically significant are shown on the path diagrams of results (Fig 2).

**Table 2:** Results of path models (sequential mediation) with rate of change in HbA_1c_ as the outcome (SSCs n = 121), healthy at baseline (no CVD/T2DM at W1), numeric predictors, standardised, and physical activity (PA), categorical (0/1), n = 2337.

Area-SES (standardised SEIFA-IEO, SSC) N = 2337	Unadjusted models			Adjusted models ^1^		
	Estimate	95% CI	P value	Estimate	95% CI	P value
*ΔHbA*_*1c*_ *on*:						
Area SES	**-0.013**	**-0.017 to -0.009**	**<0.001**	**-0.014**	**-0.018 to -0.009**	**<0.001**
MHCS	-0.002	-0.005 to 0.001	0.234	-0.002	-0.005 to 0.001	0.173
Fruit intake	0.000	-0.002 to 0.002	0.982	-0.001	-0.003 to 0.002	0.557
Vegetable intake	0.000	-0.002 to 0.003	0.863	0.000	-0.003 to 0.003	0.983
Recommended PA (vs Sedentary)	**-0.008**	**-0.014 to -0.002**	**0.012**	**-0.008**	**-0.014 to -0.002**	**0.011**
*Fruit intake on*:						
Area SES	**0.085**	**0.025 to 0.144**	**0.005**	**0.060**	**0.000 to 0.120**	**0.048**
MHCS	**0.089**	**0.043 to 0.135**	**<0.001**	**0.067**	**0.019 to 0.115**	**0.006**
*Vegetable intake on*:						
Area SES	-0.017	-0.071 to 0.036	0.525	-0.024	-0.077 to 0.029	0.376
MHCS	**0.082**	**0.026 to 0.137**	**0.004**	**0.086**	**0.033 to 0.139**	**0.001**
*Recommended PA on*:						
Area SES	**0.276**	**0.188 to 0.364**	**<0.001**	**0.224**	**0.153 to 0.335**	**<0.001**
MHCS	**0.370**	**0.259 to 0.481**	**<0.001**	**0.341**	**0.222 to 0.461**	**<0.001**
*MHCS on*:						
Area SES	**0.069**	**0.021 to 0.117**	**0.005**	0.042	-0.005 to 0.089	0.081
Indirect effects*100:						
SES-MHCS-PA	**0.585**	**0.131 to 1.039**	**0.012**	0.300	-0.063 to 0.663	0.105
SES-MHCS-Veg	**0.567**	**0.026 to 1.109**	**0.040**	0.361	-0.099 to 0.822	0.124
SES-MHCS-Fruit	**0.620**	**0.067 to 1.173**	**0.028**	0.280	-0.111 to 0.670	0.160
SES-MHCS-Diet (Fruit and Veg)	**1.187**	**0.204 to 2.170**	**0.018**	0.641	-0.160 to 1.442	0.117
SES-PA-ΔHbA_1c_	**-0.049**	**-0.089 to -0.010**	**0.015**	**-0040**	**-0.078 to -0.003**	**0.035**
SES-MHCS-PA-ΔHbA_1c_	-0.005	-0.010 to 0.001	0.079	-0.002	-0.006 to 0.001	0.182
SES-Veg-ΔHbA_1c_	0.000	-0.005 to 0.004	0.868	0.000	-0.007 to 0.007	0.983
SES-MHCS-Veg-ΔHbA_1c_	0.000	-0.001 to 0.002	0.863	0.000	-0.001 to 0.001	0.983
SES-Fruit-ΔHbA_1c_	0.000	-0.019 to 0.019	0.982	-0.004	-0.018 to 0.010	0.582
SES-MHCS-Fruit-ΔHbA_1c_	0.000	-0.001 to 0.001	0.982	0.000	-0.001 to 0.000	0.578
SES indirect effect through PA	**-0.054**	**-0.097 to -0.010**	**0.015**	**-0.043**	**-0.082 to -0.003**	**0.035**
SES indirect effect through Veg	0.000	-0.004 to 0.003	0.873	0.000	-0.006 to 0.006	0.983
SES indirect effect through Fruit	0.000	-0.020 to 0.021	0.982	-0.004	-0.019 to 0.011	0.580
SES indirect effect through Diet (Fruit and Veg)	0.000	-0.021 to 0.021	0.997	-0.004	-0.020 to 0.012	0.600
MHCS indirect effect through PA	**-0.065**	**-0.121 to -0.009**	**0.022**	**-0.058**	**-0.113 to -0.002**	**0.042**
MHCS indirect effect through Fruit	0.000	-0.020 to 0.020	0.982	-0.004	-0.020 to 0.011	0.566
MHCS indirect effect through Veg	0.002	-0.020 to 0.024	0.864	0.000	-0.023 to 0.024	0.983
MHCS indirect effect through Diet (Fruit and Veg)	0.002	-0.026 to 0.030	0.880	-0.004	-0.033 to 0.024	0.774
*MHCS total indirect effect*	**-0.063**	**-0.123 to -0.003**	**0.039**	**-0.062**	**-0.121 to -0.003**	**0.041**
*MHCS total effect*	**-0.242**	**-0.537 to 0.053**	**0.108**	**-0.272**	**-0.571 to 0.028**	**0.075**
*Total indirect effect (SES on ΔHbA*_*1c*_*)*	**-0.054**	**-0.100 to 0.008**	**0.015**	**-0.047**	**-0.089 to -0.005**	**0.030**
*Total effect (SES on ΔHbA*_*1c*_*)*	**-1.385**	**-1.791 to -0.979**	**<0.001**	**-1.399**	**-1.800 to -0.998**	**<0.001**
Model fit	AIC 22430.637	BIC 22591.822	BIC_adj_ 22502.861	AIC 21789.326	BIC 22096.750	BIC_adj_ 21893.409

^1^ adjusted for individual-level age, sex, employment status, education, marital status, and smoking status; Abbreviations: AIC: Akaike’s Information Criterion; BIC: Bayesian Information Criterion; BIC_adj_, sample size adjusted Bayesian Information Criterion; CI, confidence interval; Fruit: fruit intake (serves); HbA_1c_: glycosylated haemoglobin; ΔHbA_1c_: rate of change in glycosylated haemoglobin; MHCS: Mental Health Component score (SF-36); PA: Physical Activity; SES, Socio-Economic Status; SEIFA-IEO, Socio-Economic Index for Areas, Index for Education and Occupation; SSC: State Suburb; Veg: vegetable intake (serves). Note: Indirect effects have been multiplied by 100 for ease of presentation.

In the unadjusted (no covariates) sequential path model (Fig 2A, Table 2), area SES was directly associated with mental/emotional health (β 0.069 [0.021, 0.117], p<0.01), fruit intake (β 0.085 [0.025, 0.144], p<0.01), physical activity (meeting recommendations; β-log odds 0.276 [0.188, 0.364], p<0.001), and rate of change in HbA_1c_ (β -0.013 [-0.017, -0.009], p<0.001). 1SD greater area SES was associated with a 0.069SD greater mental health (1SD = 9.96 SF-36 MHCS points) and 0.085 greater servings of fruit (1SD = 1 serve of fruit). Similarly, 1SD greater area SES was associated with a 0.276 greater likelihood [β-log odds] of meeting physical activity recommendations and a lesser rate of increasing HbA_1c_ per year (0.013% points less than the average of 0.035% points, i.e. a 37% reduction).

Area SES was not associated with vegetable intake. Mental/emotional health was directly associated with fruit (β 0.089 [0.043, 0.135], p<0.001) and vegetable (β 0.082 [0.026, 0.137], p<0.01) intakes, and meeting physical activity recommendations (β-log odds 0.370 [0.259, 0.481], p<0.001) but was not directly associated with change in HbA_1c_. Meeting physical activity recommendations was associated with rate of change in HbA_1c_ (β -0.008 [-0.014, -0.002], p<0.05), but fruit and vegetable intakes were not statistically significantly associated with change in HbA_1c_. There were indirect effects from area SES through mental/emotional health to health behaviours (physical activity β_IE_ x100 0.585 [0.131, 1.039], p<0.05; fruit intake β_IE_x100 0.620 [0.067, 1.109], p<0.05; vegetable intake β_IE_x100 0.567 [0.026, 1.109], p<0.05; total diet β_IE_x100 1.187 [0.204, 2.170], p<0.05), but only the pathway through physical activity resulted in a statistically significant change in HbA_1c_ (area SES total indirect effect through physical activity β_IE_ x100–0.054 [-0.097, -0.010], p<0.05).

A similar pattern remained after adjustment for individual-level covariates (Fig 2B), but with attenuation of associations. Area SES was no longer statistically significantly associated with mental/emotional health and, consequently, area SES was no longer indirectly linked to health behaviours through mental/emotional health. Mental/emotional health remained associated with health behaviours (meeting physical activity recommendations β-log odds 0.341 [0.222, 0.461], p<0.001; fruit intake β 0.067 [0.019, 0.115], p<0.01; vegetable intake β 0.086 [0.033, 0.139], p<0.05) and indirectly linked to change in HbA_1c_ through physical activity (β_IE_x100–0.058 [-0.113, -0.002], p<0.05). Area SES remained directly associated with change in HbA_1c_ (β -0.014 [-0.018, -0.009], p<0.001) and indirectly linked to change in HbA_1c_ through physical activity (β_IE_x100–0.043 [-0.082, -0.003], p<0.05).

## Discussion

This study assessed the longitudinal relationships between area SES, mental health, fruit and vegetable intakes, physical activity, and change in HbA_1c_ for a 10-year cohort of residential dwelling adults in Adelaide, South Australia. For this sample and region, greater area SES was associated with more healthful diet (fruit intake) and greater physical activity and was directly *and* indirectly (through physical activity) protective against worsening HbA_1c_ (i.e., partial mediation). Greater mental health was similarly associated with more healthful dietary intake (both fruits and vegetables) and was indirectly protective against worsening HbA_1c_ through physical activity (complete mediation). Area SES was only associated with mental health in models unadjusted for individual covariates.

Numerous cross-sectional studies have reported greater area SES as inversely associated with CMR [24, 38]. Longitudinal studies, however, are less common and their evidence somewhat equivocal, though generally consistent with cross-sectional findings [24, 38]. This study importantly adds weight to the few existing longitudinal studies, providing evidence that supports causal inference through temporal ordering of measurements. Importantly, inference regarding the biological plausibility of causal relations was supported by empirically assessing mediation pathways (mental health, diet and physical activity behaviour) presumed to link area SES to HbA_1c_.

Unexpectedly, greater area SES was not associated with greater mental health–one potential mediating mechanism influencing HbA_1c_ –in models including individual covariates. A recent review reported 83% of included studies found associations between low area SES and poor mental health [32] though the findings of an older review were more equivocal [33]. Our findings suggest that individual-level factors that relate to mental health may also relate to area of residence and consequently explain the apparent association in unadjusted models (i.e., potential confounding by individual SES). For example, individuals residing in low SES areas are likely to have low individual SES and other research has reported low individual SES is related to poor mental health [8]. Though mental health did not function as a mediator between area SES and change in HbA_1c_, greater mental health was associated with more positive health behaviour (diet and physical activity) which aligns with previous literature [20, 70]. Interventions aiming to improve or support mental health may also assist interventions targeting health behaviour.

Physical activity functioned as a mediator linking both mental health and area SES to change in HbA_1c_. This finding provides empirical support for the oft-assumed mechanism of physical activity behaviour linking both area SES and individual mental health with cardiometabolic outcomes. There was no such support for dietary behaviour (fruit and vegetable intake) as a link between mental health or area SES and change in HbA_1c_. Though area SES was positively associated with fruit intake, consistent with some [27, 71, 72] but not all studies [29, 30, 71], fruit and vegetable intake was not associated with change in HbA_1c_. This result ran counter to expectations. Numerous studies have reported positive effects of healthful diet on health outcomes (for a review see [11]). Our lack of findings may reflect that fruit and vegetable intakes are often not well self-reported due to inaccurate recall, misunderstanding of questions and serving sizes, and social desirability bias [73, 74]. In addition, fruit and vegetable intake is but a component of overall diet, and other dietary factors can positively or negatively affect CMD and CMR, for example, fatty acids (PUFAs) [75], trans fatty acids [76], nuts [77], and whole grain intake [78]. The lack of association between fruit and vegetable intake and change in HbA_1c_ could also reflect an insufficient follow-up period to detect a statistically significant effect of diet on change in HbA_1c_.

Overall, these findings suggest the need to provide additional support for positive health behaviour in more deprived areas and amongst individuals with poorer mental health. Studies indicate that area SES covaries with other environmental factors. For example, studies have reported a greater availability of fast food and poorer availability of healthful food in deprived compared to less deprived areas internationally [79]. Future studies should include local environment attributes that covary with SES to examine mediation pathways between environments and health outcomes. Given that physical activity partially mediated the relationship between area SES and change in HbA_1c_, empirical assessment of pathways from other environmental factors that may support (e.g., walkability, public open space) or inhibit (e.g., lack of safety) physical activity and thus influence health outcomes, is recommended. One recent report of this same cohort indicated that physical activity partially mediated associations between walkability, local descriptive norms for overweight/obesity, local descriptive norms for physical inactivity, and change in HbA_1c_ [80]. Our findings of mediation through physical activity but not through diet suggests that residential environmental features supporting or inhibiting physical activity may be more important in relation to cardiometabolic outcomes than features relating to dietary intakes.

There remained a direct effect from area SES to change in HbA_1c_ after accounting for physical activity and diet as potential mediators. This supports the premise of a direct non-cognitive pathway linking poor area SES to CMR through allostatic loading as a response to non-perceived chronic stress [36]. Consequently, interventions focusing on improving individual health behaviours are unlikely to completely remove health disparities that exist between areas, even when such interventions do develop environments that are supportive of healthful behaviours such as improving area walkability and access to public open space. Improvements to areas that reduce the non-perceived chronic stress of residents will also be needed. Interventions could target social stressors embedded within more deprived areas such as social disorder. Efforts should also seek to reduce social and economic inequalities between areas.

Though a body of research has assessed relationships between key built and social environmental factors and diet, physical activity and CMR, few studies have empirically tested mediation pathways or been longitudinal in design. More such studies are needed. Environments, behaviours and health outcomes are features of complex systems with multiple exposures, pathways, and interacting factors collectively contributing to health outcomes. The findings of this study support the premise of complexity within these systems, finding complete mediation between mental health and change in HbA_1c_ through physical activity and partial mediation between area SES and change in HbA_1c_ through physical activity. This partial mediation suggests there may be other mediating factors not assessed in this study, but also provides support for adverse physiological responses within a non-cognitive pathway linking area SES directly to change in CMR.

## Strengths and limitations

The longitudinal design of this study is a strength, with area exposure occurring prior to assessment of mental health and behaviours, and the main outcome being expressed as rate of change in HbA_1c_ over time. This temporal ordering of measurement supports causal inference [39]. Additionally, the empirical assessment of biologically plausible mechanisms linking area SES to HbA_1c_ provides further support for causal inference [39]. The outcome, HbA_1c_, was clinically measured, avoiding problems known to be related to self-report. The potential for self-report bias was not completely avoided however, as mental health, physical activity, and dietary intake information were self-reported. Use of other measures such as pedometer or accelerometer collected physical activity information, data from food diaries or a more detailed diet questionnaire, or mental health assessed by a psychologist could provide more reliable and valid assessments of these variables. Such data were not available within the context of the NWAHS cohort. Use of improved mental health and behavioural measures is recommended where possible within future studies. However, the survey questions used in this study to collect mental health, physical activity and diet behaviour information have previously demonstrated acceptable validity and reliability and have been used in other Australian research and for population health surveillance. Importantly, the error variance introduced from the self-report measures used here is likely to have reduced the strength of associations. As such, the strength of actual relationships may be greater than reported here [11].

Area SES was expressed for State Suburbs, a pre-defined administrative unit and consequently reported findings may be influenced by the Modifiable Areal Unit Problem (MAUP) [81]. The consistency of associations between area SES and CMR across multiple studies and regions and using different expressions of area SES including different aggregations of administrative units, suggests that although the MAUP may have influenced the size of reported coefficients, it has not affected the directions of reported associations. There can be little doubt as to the positive relationship between greater area SES and health. This study has focused on participant’s residential area SES, however other commonly visited places, such as the workplace, may influence mental health, health behaviour and health outcomes [82]. In addition, residential self-selection may confound estimates of association between area exposure and health behaviour or outcome. However, this bias is likely not very strong, typically attenuating estimated effects rather than rendering them no longer statistically significant [83]. The NWAHS cohort is broadly representative of the Adelaide population and as such, the findings of this study should be generalisable to populations of similar urban residential environments.

## Conclusion

This study provides empirical support for physical activity as a partial mediator accounting for the association between area SES and adverse changes in HbA_1c_. Additionally, physical activity completely mediated associations between mental health and change in HbA_1c_. Findings did not indicate mediation from area SES to change inHbA_1c_ through mental health or dietary intakes. Area SES may, however, also influence HbA_1c_ directly through a non-cognitive stress-related response to adverse environmental factors.

Disadvantaged areas and individuals with poorer mental health should be targeted for interventions aiming to improve population cardiometabolic health. Though diet is undoubtedly important to health, the findings of this research suggest that area SES and individual mental health influence HbA_1c_ predominantly through physical activity as opposed to fruit and vegetable intakes. Attention to developing physical activity supportive environments is recommended along with other physical activity promotion strategies. Interventions focused on improving mental health may have flow on effects and improve physical activity and diet behaviour, and indirectly through physical activity, may improve cardiometabolic health.

## Supporting information

S1 File Table 1 results of path models (parallel mediation) with rate of change in HbA_1c_ as the outcome (SSCs n = 121), healthy at baseline (no CVD/T2DM at W1), numeric predictors, standardised, and physical activity (PA), categorical (0/1), n = 2337. (DOCX)

## Abbreviations

ΔHbA_1c_: rate of change in glycosylated haemoglobin
ABS: Australian Bureau of Statistics
AIC: Akaike’s Information Criteria
BIC: Bayesian Information Criteria
CATI: Computer-Assisted Telephone Interview
CDs: Census Collection Districts
CMD: cardiometabolic disease
CMR: cardiometabolic risk
CVD: cardiovascular disease
FIML: Full information maximum likelihood
HbA_1c_: glycosylated haemoglobin
ICC: Intraclass correlation
MAUP: Modifiable Areal Unit Problem
MHCS: mental health component score of the Short Form 36
NWAHS: North West Adelaide Health Study
OECD: Organisation for Economic Co-operation and Development
PA: physical activity
PAMS: Project Place and Metabolic Syndrome Project
PCS: physical component score of the Short Form 36
SEIFA-IEO: Socio-Economic Index for Areas, Index for Education and Occupation
SEM: structural equation modelling
SES: socioeconomic status
SF-36: Short Form 36
SSC: State Suburb
T2DM: Type 2 Diabetes Mellitus
W1: Wave 1
W2: Wave 2
W3: Wave 3
