# Supplementary material for: Associations between area socioeconomic status, individual mental health, physical activity, diet and change in cardiometabolic risk amongst a cohort of Australian adults: A longitudinal path analysis
Source: PLoS One. 2020 May 29;15(5):e0233793. doi: 10.1371/journal.pone.0233793 (PMC7259701; doi:10.1371/journal.pone.0233793)
Supplement: S1 File — (DOCX) [file pone.0233793.s001.docx]

Table 1 Results of path models (parallel mediation) with rate of change in HbA_1c_ as the outcome (SSCs n=121), healthy at baseline (no CVD/T2DM at W1), numeric predictors, standardised, and physical activity (PA), categorical (0/1), n=2337

| **Area-SES (standardised SEIFA-IEO, SSC) N=2337** | **Unadjusted models** | | | **Adjusted models ^1^** | | |
| --- | --- | --- | --- | --- | --- | --- |
|  | **Estimate** | **95% CI** | **P value** | **Estimate** | **95% CI** | **P value** |
| *ΔHbA_1c_ on:* |  |  |  |  |  |  |
| Area SES | **-0.013** | **-0.017 to -0.009** | **<0.001** | **-0.014** | **-0.018 to -0.009** | **<0.001** |
| MHC | -0.002 | -0.005 to 0.001 | 0.192 | -0.002 | -0.005 to 0.001 | 0.146 |
| Fruit intake | 0.000 | -0.002 to 0.002 | 0.973 | -0.001 | -0.003 to 0.002 | 0.556 |
| Vegetable intake | 0.000 | -0.002 to 0.003 | 0.853 | 0.000 | -0.003 to 0.003 | 0.970 |
| Recommended PA (v sedentary) | **-0.008** | **-0.014 to -0.002** | **0.011** | **-0.008** | **-0.014 to -0.002** | **0.009** |
| Fruit intake on Area SES | **0.090** | **0.030 to 0.150** | **0.003** | **0.062** | **0.002 to 0.120** | **0.042** |
| Vegetable intake on Area SES | -0.013 | -0.067 to 0.042 | 0.651 | -0.022 | -0.076 to 0.033 | 0.440 |
| Recommended PA (v sedentary) on Area SES | **0.288** | **0.198 to 0.378** | **<0.001** | **0.250** | **0.157 to 0.343** | **<0.001** |
| MHC on Area SES | **0.070** | **0.022 to 0.118** | **0.004** | 0.042 | -0.005 to 0.089 | 0.077 |
| *Indirect effects*100:* |  |  |  |  |  |  |
| SES-MHC-ΔHbA_1c_ | -0.014 | -0.037 to 0.009 | 0.239 | -0.009 | -0.026 to 0.007 | 0.263 |
| SES-PA-ΔHbA_1c_ | **-0.051** | **-0.092 to -0.010** | **0.014** | **-0.041** | **-0.077 to -0.004** | **0.031** |
| SES-Veg-ΔHbA_1c_ | 0.000 | -0.004 to 0.003 | 0.864 | 0.000 | -0.006 to 0.006 | 0.970 |
| SES-Fruit-ΔHbA_1c_ | 0.000 | -0.020 to 0.021 | 0.973 | -0.004 | -0.019 to 0.011 | 0.580 |
| SES indirect effect through Diet (Fruit and Veg) | 0.000 | -0.021 to 0.021 | 0.998 | -0.004 | -0.020 to 0.012 | 0.600 |
| *Total indirect effect (SES on ΔHbA_1c_)* | **-0.065** | **-0.115 to -0.015** | **0.011** | **-0.054** | **-0.098 to -0.011** | **0.015** |
| *Total effect (SES on ΔHbA_1c_)* | **-1.394** | **-1.802 to -0.986** | **<0.001** | **-1.406** | **-1.809 to -1.004** | **<0.001** |
| Model fit | AIC 22490.098 | BIC 22634.013 | BIC_adj_ 22554.584 | AIC 21774.984 | BIC 22126.138 | BIC_adj_ 21932.329 |

^1^ adjusted for individual-level age, sex, employment status, education, marital status, and smoking status; Abbreviations: AIC: Akaike’s Information Criterion; BIC: Bayesian Information Criterion; BIC_adj_, sample size adjusted Bayesian Information Criterion; CI, confidence interval; Fruit: fruit intake (serves); HbA_1c_, glycosylated haemoglobin; ∆HbA_1c_, rate of change in glycosylated haemoglobin; MHC: Mental Health Component score (SF-36); PA: Physical Activity; SES, Socio-Economic Status; SEIFA-IEO, Socio-Economic Index for Areas, Index for Education and Occupation; SSC: State Suburb; veg: vegetable intake (serves). Note: Indirect effects have been multiplied by 100 for ease of presentation.
